# Single-cell mass spectrometry imaging: platform advances for multimodal spatial omics

**DOI:** 10.1007/s00216-026-06644-6

**Published:** 2026-07-02

**Authors:** Lauren E. Hill, Lyndsay E. A. Young, James W. Dressman, Peggi M. Angel, Anand S. Mehta, Richard R. Drake

**Affiliations:** Department of Pharmacology and Immunology, Medical University of South Carolina, Charleston, SC 29425 USA

**Keywords:** Mass spectrometry imaging, Single-cell imaging, Spatial omics, MALDI-MSI

## Abstract

Spatial omics has transformed biomedical research by uncovering the molecular characterization of biological systems while preserving spatial context. Among these approaches, mass spectrometry imaging (MSI) provides a label-free, in situ visualization of diverse molecular classes, including metabolites, lipids, proteins, and glycans. Recent advances in instrumentation, sample preparation, and data acquisition have pushed MSI into the field of single-cell analysis, providing unprecedented access to cellular heterogeneity and molecular states across biological contexts. Here, we review current single-cell MSI platforms and highlight key innovations that have improved spatial resolution, sensitivity, and throughput. Presented examples from published workflows highlight the variability in strategies for cell isolation, capture, and data acquisition. The three main ionization techniques of desorption electrospray ionization (DESI), secondary ion mass spectrometry (SIMS), and matrix-assisted laser desorption ionization (MALDI) are highlighted for their capabilities to generate robust single-cell multi-omics profiling. We outline future directions for the field and the potential of single-cell MSI to impact translational spatial omic research and precision medicine.

## Introduction

Spatial omics has emerged as a transformative approach in the field of biomedical research by offering critical insights into the molecular heterogeneity that underlies complex biological processes and disease progression. Among spatial omics methods, mass spectrometry imaging (MSI) is a powerful technique for visualizing the molecular composition in situ. MSI preserves the native molecular architecture of samples and acquires mass spectra across a biological specimen to generate high-resolution ion intensity maps for direct visualization of the spatial distribution of specific biomolecules. Furthermore, workflow optimizations have significantly enhanced analytical sensitivity and reproducibility, facilitating robust sequential multi-omic analysis across key molecular classes such as proteins, metabolites, lipids, and glycans [1–7].

While MSI has emerged as a powerful modality within spatial omics, it is important to position it within a broader landscape of mass spectrometry–based approaches for single-cell analysis, each offering distinct advantages and limitations. As single-cell MSI techniques provide capabilities often spanning toward subcellular spatial resolution, high-sensitivity metabolite detection, and multiplexed protein quantification, these technologies differ fundamentally in their balance of spatial fidelity, molecular coverage, sensitivity, and sample preservation. Consequently, MSI should be considered one of several emerging strategies for assessment of spatially resolved molecular profiling at cellular scales, rather than a singular solution. A comprehensive understanding of these trade-offs is essential for selecting the appropriate platform and for interpreting single-cell data within the broader context of spatial and systems biology.

There are three main MSI ionization sources currently in use to achieve high-resolution single-cell data: secondary ion mass spectrometry (SIMS), matrix-assisted laser desorption/ionization (MALDI), and electrospray ionization (ESI) [8]. Together, these represent distinct but complementary strategies within the broader landscape of spatially resolved mass spectrometric approaches, each carrying its own technical strengths and practical trade-offs. The earliest approach to reach subcellular resolution was SIMS, with pioneers in the MSI field mapping small molecules and lipids within individual neurons and cell membranes [9]. The use of MALDI for MSI analysis of tissues was first reported in 1997 [10]. Until around 2010, most MALDI-MSI studies were typically using 100–200 um spatial resolutions due to the instrumentation and software available, and had limited matrix application options and long acquisition times [11]. In the subsequent 16 years, major advancements in instrumentation, software, and sample preparation are such that commercial instruments can routinely achieve 5 μm spatial resolution, while evolving state-of-the-art instruments can reach 1 μm and lower resolutions at the single-cell level [12]. Further advancements in MALDI-MSI utilize a laser microprobe smaller than the cells themselves, allowing for distinct chemical and biological mapping of subcellular and intracellular environments [13, 14].

In the sections that follow, we review the latest innovations in single-cell MSI, highlighting strategies for sample preparation, acquisition methods, and emerging approaches in data processing and analytics. MSI is one of several mass spectrometric platforms now enabling spatially resolved molecular profiling at cellular scales; by surveying current progress across these platforms, we aim to provide an overview of the evolution of methods while highlighting the particular strengths of MALDI-MSI, its reproducibility, sensitivity, and scalability, relative to alternative approaches. We conclude by predicting future directions of the field, with emphasis on the emergent use of these platforms for profiling circulating cellular markers.

## Single-cell MSI techniques

As MSI-based technologies have advanced, increasing attention has turned toward adapting these platforms for direct single-cell analysis. However, achieving this level of resolution requires strategies capable of preserving spatial fidelity while maintaining sufficient sensitivity. Single-cell-based MS analysis can be divided into various categories based on features such as detection strategy, component analysis and imaging analysis.

SIMS has been able to image cells with lateral resolution as low as 30–50 nm while maintaining high spatial resolution for single-cell imaging [15, 16]. This instrument uses a continuous focused beam of ions to remove material from the surface of a sample by sputtering. SIMS is often coupled with time-of-flight (TOF) mass analyzers, allowing for ions with the same energy but different masses to be separated via travel time, ultimately increasing the sensitivity and mass range. TOF-SIMS was first used to image small molecule compounds in unicellular protozoa [17]. Further advancements in TOF-SIMS such as the incorporation of an Orbitrap mass spectrometer, otherwise known as 3D OrbiSIMS, has also been shown to increase the mass resolution and accuracy down to the single-cell level [18, 19]. Although various workflows demonstrate the Orbitrap’s MSI capabilities on a cellular level, the workflow is low throughput due to acquisition time, for only < 20 cells were examined in the workflow [19]. Other SIMS techniques such as nanoscale secondary ion mass spectrometry (NanoSIMS) methods have also been utilized for single-cell methods, and these often require the use of isotope or antibody labeling for analysis [20, 21].

Using metal-conjugated tags for imaging has proven particularly powerful for interrogating cellular phenotypes and microenvironment interactions within complex tissues, including immune infiltrates in tumors and spatially organized niches [22, 23]. These approaches achieve submicron resolution, allowing for precise localization of proteins to cellular compartments such as membranes, and nuclei, as well as providing insights into cell-cell interactions and functional states that are difficult to resolve by other adjacent imaging approaches [24, 25]. Because metal isotopes are not subject to photobleaching or autofluorescence, high signal stability across long acquisition times allows for consistent quantification across large imaging areas [26, 27].

An additional strength of metal-tagged approaches is that it is inherently targeted by antibodies and allows focused spatial analysis of specific cell populations [28]. Although this requires prior knowledge of relevant markers, validated antibody panels are available for assays on cell and morphological composition, thereby occupying a critical niche in the single-cell spatial omics landscape that can be integrated with untargeted MSI datasets. Emerging multimodal workflows increasingly combine metal-tagged-based immunophenotyping with MALDI-MSI or other spatial metabolomic platforms, enabling direct alignment of cellular identity with endogenous molecular states and reinforcing the value of these as complementary tools rather than a standalone solution for comprehensive single-cell spatial profiling [29].

Multiplexed ion beam imaging (MIBI) represents a distinct but highly complementary class of single-cell spatial analysis that extends SIMS-based approaches toward targeted, antibody-driven proteomic mapping. Unlike conventional SIMS or NanoSIMS, workflows that primarily focus on endogenous ions or isotopically labeled metabolites, MIBI leverages antibodies conjugated to stable metal isotopes to enable highly multiplexed, quantitative imaging of proteins at subcellular resolution. A primary ion beam rasterizes across the sample surface, releasing metal tags that are subsequently detected as secondary ions by time-of-flight mass spectrometry. Because each antibody is labeled with a unique metal isotope, MIBI avoids spectral overlap common to fluorescence-based imaging and enables simultaneous detection of 40–50 protein targets within a single tissue section or cell population [26].

Metal-tag-based imaging approaches such as cytometry by time‑of‑flight (CyTOF), imaging mass cytometry (IMC), and MIBI are widely used platforms for multiplexed single-cell analysis in tissue [26, 30]. CyTOF effectively integrates inductively coupled plasma ionization with time-of-flight mass spectrometry to quantify antibodies conjugated to isotopically pure heavy-metal tags. Imaging mass cytometry (IMC) extends the CyTOF principle to tissue sections in order to preserve spatial context at subcellular resolution [31, 32]. Because each metal isotope occupies a discrete mass channel with minimal spectral overlap, these methods enable simultaneous quantification of approximately 40–60 protein markers per cell with high sensitivity. Cellular resolution in these workflows is achieved through segmentation of intact cellular structures within preserved tissue architecture, rather than relying solely on reduction of pixel size [33]. These results enable specific neighborhood analysis for robust mapping of various immune cell interactions within a microenvironment, making IMC modalities widely adopted in spatial immunology and tumor microenvironment studies. Similar approaches, such as multiplexed ion beam imaging (MIBI), utilize this metal-isotope-tagged antibody approach, while coupling it ion beam–based desorption and SIMS. Furthermore, coupled with a TOF analyzer, MIBI achieves subcellular imaging that is both high throughput and highly multiplexed, without compromising sensitivity. Furthermore, fluorescence in situ hybridization (FISH) SIMS is another hybrid, a targeted technique that utilizes correlative microscopy for single-cell imaging analysis [34, 35].

Alternatively, there are label-free, untargeted techniques that achieve high sensitivity using SIMS modalities. Due to its high vacuum requirements and low ionization efficiency of biomolecules, SIMS technologies can achieve submicron spatial resolution for detection of various elemental analyses, isotope tracing experiments, and certain classes of lipids and metabolites [36, 37]. For TOF-SIMS, a full mass spectrum is acquired at each pixel, enabling spatial mapping of multiple molecular classes. These approaches are highly sensitive, as TOF-SIMS has been used in many different studies to trace drug delivery along with its various multi-omic profiling workflows [17, 38–40].

ESI-based imaging techniques have also been used for single-cell MSI, with the most widely used approach being DESI-MSI, which is a spray-based ionization method where a charged solvent is applied onto the sample surface, and the ion beam desorbs and ionizes the analyte molecules [41]. However, these droplets are too large to resolve an individual cell using standard DESI, thus nanospray desorption electrospray ionization (nano-DESI) has been applied to profile single cells via use of secondary capillary to transport desorbed analytes [42, 43]. Other recently published DESI platforms include ultralow-flow-rate desorption electrospray ionization mass spectrometry imaging (u-DESI-MSI), which utilizes the dramatic reduction of the solvent flow rate to create a focused spray spot on the sample surface, leading to a smaller “pixel” size to achieve subcellular resolution [44]. Various groups have demonstrated nano-DESI’s metabolomic and lipidomic single-cell abilities; however, these methods have shown to be limited in their throughput and sensitivity [40]. Furthermore, laser ablation electrospray ionization (LAESI) is an ESI-based technique which involves an infrared (IR) laser to ablate the molecules from the surface, although lower throughput, capturing only about 100 cells per hour, and current work is focused on achieving the sensitivity necessary for single-cell analysis [45]. It is expected that continued advancements in ESI involving microfluidic platforms, droplet-based sample handling, and microscale chromatography will help to overcome current throughput bottlenecks for these ESI sources to achieve the high-resolution, high-sensitivity results [46].

MALDI-MSI has seen significant growth in the context of single-cell spatial omic analysis over the last 25 years [47]. MALDI involves application of an organic chemical matrix directly to the sample to assist in the ionization of laser desorbed biomolecules. The matrix aids in the energy transfer processes from a pulsed laser beam to the sample, for upon irradiation, the matrix absorbs the laser energy and transfers it to the surrounding analytes, causing their desorption into the gas phase. The reactions between the matrix and embedded biomolecules facilitate soft ionization, enabling intact molecular ions to be generated with minimal fragmentation. This method allows for targeting a dynamic mass range while also achieving a lateral resolution down to 1–5 µm to allow for sensitive, high-resolution in situ analysis. Initially, methods to achieve single-cell analysis by MALDI-MSI used cultured cells attached to glass slides, enabling individual cells to be spatially isolated and uniformly matrix-coated for accurate single-cell interrogation [48]. In one example, reproducible, low-density cell culture arrays have shown the capability to identify complex N-glycans with high sensitivity in multiple cell lines co-registered to single cells [49]. Isotopic detection of aminosugars with glutamine (IDAWG), a method that allows precise measurement of N-glycan turnover rates and synthesis dynamics by incorporating heavy nitrogen tags directly into cellular aminosugars [50].

MALDI-MSI has found broad application in the field of single-cell molecular profiling due to its ability to obtain non-targeted, highly specific multi-omic information while still prioritizing sample processing efficiency. Li and colleagues have demonstrated reproducible single-cell metabolomics and lipidomics at the single-cell scale [51]. Others have shown single-cell resolution capabilities via coupling MALDI with various techniques, such as immunohistochemistry (MALDI-IHC), introducing the power and multiplex capability of the platform [7, 52, 53]. A recent development in MALDI imaging includes the addition of a second laser following initial desorption termed MALDI-2. Secondary ionization has also shown to be a promising technique for single-cell analysis, as it allows researchers to detect a wider range of molecules and those present at lower concentrations [54, 55]. With MALDI-2, oversampling techniques have also been shown to enhance detectability of low-intensity samples and low-abundance species [56]. Other variations in instrumentation include the usage of atmospheric pressure MALDI (AP-MALDI), which unlike traditional MALDI, occurs in open air and not under vacuum, often simplifying sample prep and handling [57]. Transmission-mode MALDI (MALDI) is designed to improve lateral resolution by employing a “through-sample” geometry in which the laser is focused through the backside of the substrate on the slide, rather than the front, thereby allowing higher-numerical aperture optics and smaller ablation craters. Recently, combinations of t-MALDI with laser post-ionization (MALDI-2) have improved sensitivity while retaining < 1 µm resolution in tissue sections and detection of up to 200 lipids and nucleotides [14]. Strategies to complement these techniques include dual-polarity MALDI which acquires data in both positive and negative ion modes at the same coordinates so that lipids and metabolites with different ionization propensities are captured. Recent work has shown that dual-polarity acquisition, especially when coupled to ion mobility or trapped-ion separation, increases detected lipid species at cellular resolution without requiring repeated matrix re-applications [51, 58]. A summary of sample techniques and notable representative examples for single-cell MSI is presented in Table 1.

**Table 1:** Summary of single-cell MSI techniques

Single-cell MSI method	Sensitivity/resolution	Molecular coverage	Mass range (m/z)	Sample throughput (cells)	Sample prep	Limitations	References/notes
SIMS	30–100 nm	Metabolites, lipids	Broad (typically 100–1000)	~ 30 cells	Extensive	Surface damage, limited depth profiling	[18]
OrbiSIMS (3D)	300 nm	Metabolites, lipids	100–1000	< 20 cells	Extensive	Low throughput due to acquisition time	[20]
NanoSIMS	50 nm	Isotopic species	100–2000	Variable	Extensive	Requires isotopic or antibody labeling	[21, 22]
MIBI-TOF	300 nm	Proteins (via metal tags)	Variable	Low	Extensive	Requires exogenous metal isotopic tags	[24–27]
DESI	20–100 µm	Metabolites, lipids	100–1000	Moderate	Minimal	Droplet size too large for single-cell	[28]
Nano-DESI	~ 10 µm	Metabolites, lipids	100–1200	Low	Moderate	Low throughput, reduced sensitivity	[29, 30]
u-DESI-MSI	Subcellular (< 5 µm pixel)	Metabolites, lipids	100–1200	Low	Moderate	Limited reproducibility; still emerging	[31]
LAESI	~ 30 µm	Metabolites	100–1000	~ 100 cells/hour	Moderate	Low throughput and low sensitivity	[33]
MALDI	1–5 µm	Metabolites, lipids, peptides	Up to > 3000	High	Moderate	Matrix application can affect reproducibility	[4, 38]
MALDI-IHC	1–5 µm	Proteins, metabolites	Broad	High	Extensive (antibody + matrix)	Requires validated antibodies	[39]
MALDI-2	1–2 µm	Metabolites, lipids, peptides	Broad	Moderate	Moderate	Oversampling required; complex setup	[41, 42]
AP-MALDI	5–10 µm	Metabolites, lipids	Broad	High	Simple (no vacuum)	Lower sensitivity than vacuum MALDI	[44]
t-MALDI (transmission mode)	< 1 µm	Lipids, nucleotides	Up to 200 species detected	Low	Moderate	Requires specialized geometry	[45]
Dual-polarity MALDI	1–5 µm	Lipids, metabolites	Broad	Moderate	Moderate	Requires advanced instrumentation	[38]

## Table of SC MSI methods (attached)

While this review emphasizes MALDI-MSI, it is important to contextualize this platform within the field, as various approaches share MSI as a detection strategy but differ fundamentally in whether measurements are targeted (antibody-based) or untargeted (label-free molecular profiling). Although these modalities share the common principle of coupling spatially resolved sampling with mass spectrometry, they differ substantially in their analytical strategies, molecular targets, and mechanisms to achieve cellular resolution. While some platforms rely on detection of predefined protein markers, others employ label-free ionization of endogenous biomolecules. Consequently, interpreting the capabilities of any individual platform requires consideration within this broad framework in terms of achieving validated cellular resolution, architectural preservation, or multi-omic capabilities.

IMC offers images at high resolution via targeted approaches; the field of SIMS has advanced in its capabilities of achieving both targeted and untargeted single-cell workflows, thereby offering opportunities for downstream analysis. In comparison to MALDI-MSI, these platforms use laser ablation and mass spectrometric detection to achieve validated cellular resolution while maintaining cellular neighborhoods and tissue architecture in ways that do not rely on pixel size. MALDI-MSI single-cell workflows often depend on careful control of image correlation and resolution, as well as downstream computational approaches. In order to achieve similar resolution to IMC and SIMS, MALDI-MSI techniques like laser post-ionization (MALDI-2) and oversampling are often necessary to enhance signal [54]. For spatial omics applications, however, MALDI excels in its multi-omic molecular profiling workflows, particularly for metabolic, lipidomic, or glycomic profiling often on the same tissue [5, 7]. SIMS-based workflows are highly destructive to tissue and cell targets, limiting multiplexed options on the same sample slide [48, 59].

Recently, various approaches to improve data integration and single-cell imaging include the optimization of both MALDI-MSI and imaging mass cytometry (IMC) allowing for both targeted and untargeted single-cell analysis in situ [31]. This strategy is favorable in its ability for cellular composition analysis alongside direct quantification of metabolic phenotypes.

## Trade-offs in single-cell mass spectrometry imaging

In the field of MSI, achieving single-cell resolution across imaging modalities requires balancing sample preparation, ionization efficiency, and molecular coverage. Additionally, as each platform has its own unique workflow and acquisition method, there are fundamental resolution–sensitivity trade-offs associated with each. A rigorous interpretation of single-cell MSI data therefore requires the evaluation of ion yield, detectable analyte counts, and feasibility of the workflow. With single-cell MALDI-MSI, as pixel and laser spot size decrease, the sampled area and consequently the total number of desorbed/ionized molecules decrease, and ion counts may fall below the detection threshold. As quantitative robustness is reduced, and consequently distinguishing biological heterogeneity from technical noise becomes challenging, strategies to mitigate sensitivity without decreases in resolution include using a laser step size smaller than the laser spot diameter as well as post-ionization approaches such as MALDI-2. Choice of chemical matrix used and how it is applied to the sample are also considerations.

In MALDI workflows that focus on the analysis of various molecular classes such as N-glycans, lipids, and metabolites, additional sample preparation and analytical challenges exist. Analysis of released N-glycans requires direct enzymatic digestion that can introduce variability in efficiency and spatial localization. Similarly, lipid and metabolite detection is highly sensitive to matrix selection and application, and ion suppression across these single-cell platforms remains a challenge. Collectively, these factors highlight the need for standardized sample preparation protocols, appropriate reference materials, and careful normalization strategies to improve quantitative reliability.

SIMS-based imaging achieves high spatial resolution with sensitivity at the submicron range via highly focused ion beams, but this at the cost of a smaller sampled volume and limits in fragmentation. Recent advances such as Orbitrap-SIMS or NanoSIMS improve molecular specificity and detection, but sensitivity remains tightly coupled to ionization efficiency much like MALDI-MSI [19, 21, 27]. NanoSIMS provides high resolution due to utilization of a finely focused primary ion beam sputtering secondary ions from the sample surface, but is limited in its small sampling volume. Recent developments in SIMS include correlated light and electron microscopy-SIMS (CLEM-SIMS) which integrates SIMS modalities with ultrastructural imaging from transmission electron microscopy [60]. Cells are prepared as ultrathin sections and electron microscopy provides nanometer-scale structural context of organelles and membranes while simultaneous data acquisition with SIMS is performed to map chemical or isotopic composition. This approach enables high resolution and sensitivity but is limited in its low-throughput design and decreased sample volume for analyte detection due to the ultrathin sectioning.

ESI single-cell approaches have increased detection of low-abundance species such as metabolites and lipids with higher sensitivity per cell; however, this is often at the cost of loss of spatial architecture and decreases in throughput. New improvements in the spatial resolution of DESI-MSI include use of nano-DESI with microscopy assistance to reach ten-micron resolution [61]. Other techniques such as DEFFI, STC-DESI, u-DESI, and t-SPESI share a common principle of localized solvent-mediated extraction of analytes followed by electrospray ionization [44, 62–64]. Unlike MALDI or SIMS, these approaches operate under ambient conditions and avoid matrix deposition or high-energy sputtering, which can better preserve metabolites. DESI workflow trade-off lies in its workflow, as decreasing the solvent interaction area reduces total extracted material, but this is offset by the relatively high ionization efficiency.

Targeted imaging platforms, including IMC and MIBI, achieve high sensitivity and ~ 1 µm resolution while preserving tissue architecture. Sensitivity is governed by epitope abundance and antibody binding, rather than endogenous molecular concentrations, thereby enabling robust single-cell quantification. IMC’s optical immunohistochemistry lies in its high multiplexing capacity that has shown greater sensitivity, specificity, and feasibility, but these approaches are restricted to predefined protein targets and require fixed tissue sections, limiting applicability to suspension and in vitro-based assays. Conceptually, IMC allows for highly robust single-cell segmentation and preserved spatial architecture. Similar in concept, immunofluorescence-guided MALDI workflows that use photocleavable mass tags offer greater molecular extensibility and untargeted MSI, but have lower sensitivity and stronger dependence on ionization efficiency [7, 53]. It is expected that continued focus on data integration of spatial omics using MALDI and immunophenotyping by IMC will advance the field of single-cell MSI by merging each of their unique strengths within resolution, sensitivity, and robustness [31].

## Cell capturing and sample prep techniques

**Fig. 1:**
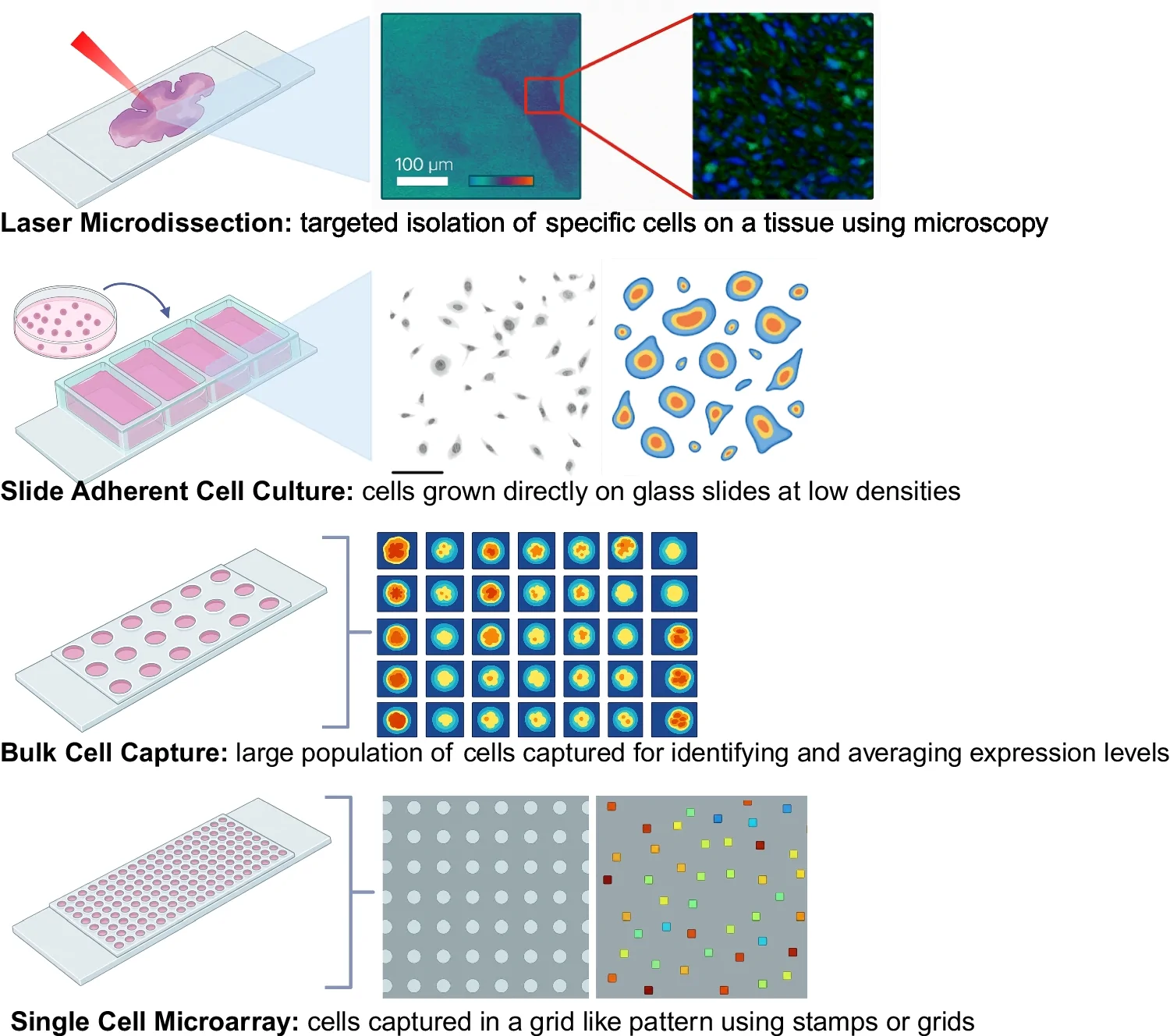
Representative sample preparation approaches. *Laser microdissection* enables targeted excision of defined regions of individual cells from tissue sections using microscopy-guided laser cutting. *Adherent cell culture slide* uses a low concentration of cells captured on slides and involves culturing adherent cells followed usually by microscopy imaging to identify cell coordinates for proper targeting during MSI. *Bulk cell capture* involves spotting 1 µl spots of thousands of cells onto coated slides to measure an average molecular signal across many cells, but at the loss of single-cell resolution. *Single-cell microarrays* use patterned grids or stamps to capture individual cells in defined locations, facilitating high-throughput single-cell analysis with spatial organization. These approaches span a range of spatial resolution, throughput, and cellular specificity

Sample preparation is a critical component for single-cell MSI, as the data quality relies heavily on key components such as throughput, sensitivity, reproducibility, and cellular integrity. Furthermore, single-cell isolation and sample preparation is a highly intricate and multi-layered process, and workflow design across published techniques varies significantly. As summarized in Fig. 1, methods for isolation of single cells include low-density cell culturing directly on slides, microarrays, laser capture micro-dissection (LCM), capillary-based sampling, and fluorescence-guided techniques [55, 65–67]. Cell types vary throughout workflows, for adherent cells are accessible due to their ability to bind to the slide, while suspension cells such as lymphoma cell lines or clinical peripheral mononuclear cells offer much more clinically relevant opportunities for MALDI-MSI utilization. Fluorescence-activated cell sorting (FACS) and flow cytometry can be integrated as preparative steps prior to MSI analysis; however, these can alter the cellular surface thereby limiting detection of some metabolite species [38]. Systems using micro-pipetting or capillary-based approaches involve extraction of the single cell from a sample surface, typically a bulk cell or bulk tissue sample [67, 68]. Microdissection (LCM) provides a direct approach for removing and isolating individual cells within tissue sections for subsequent analysis [69].

For MALDI, DESI, and SIMS methods, one of the most common approaches is low-density culturing directly on conductive ITO-coated glass slides. In this approach, cells are seeded sparsely to ensure physical separation and allowed to adhere before fixation or matrix application. One method to improve consistency for adherence of cells to a slide is the use of predefined micropatterned substrate arrays which work to confine cell adhesion to defined positions [70]. For each cell, the goal is to extract a single representative mass spectrum by combining the signal from all image pixels that correspond to that cell’s physical location. This can be done manually, by selecting the pixels visually, or more systematically by co-registering the MSI data with corresponding microscopic images of the same area [47].

## Current single-cell MSI acquisition methods

**Fig. 2:**
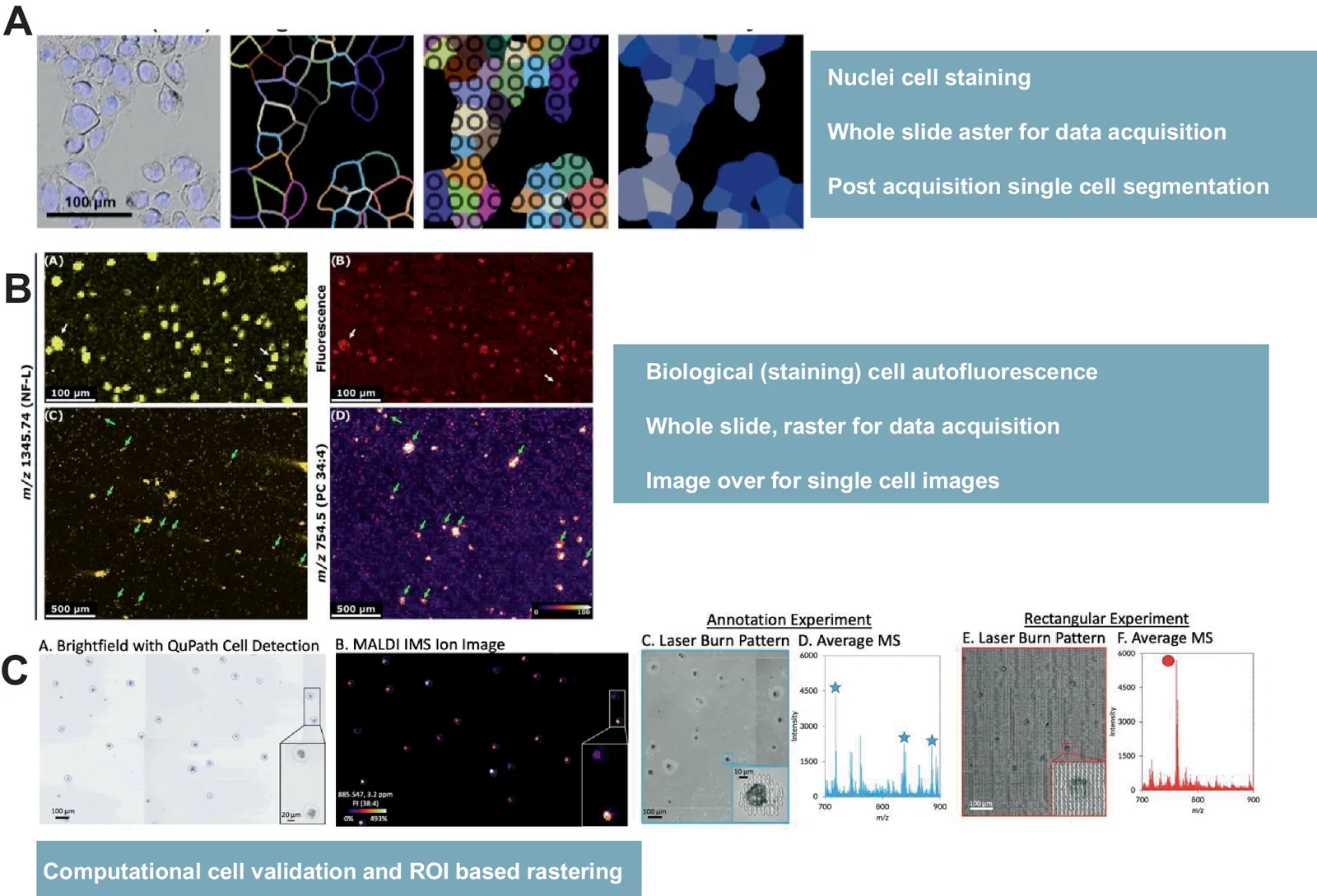
Representative strategies for single-cell image acquisition in MALDI-MSI. **A** Post-acquisition cell segmentation approach exemplified by HT SpaceM [73]. Nuclei staining (left) enables cell identification prior to a whole-slide, uniform-raster MALDI-MSI acquisition. **B** Multimodal fluorescence-guided acquisition demonstrated in glioblastoma patient-derived cells [53]. **C** Microscopy-driven region-of-interest (ROI)–based acquisition [74]. Figures adapted with permission

Single-cell MSI workflows have highlighted the need for optical images to guide cell segmentation and transferred them onto the MS images [71]. Unlike bulk MSI, detection across large tissue areas, single-cell workflows must assign each molecular spectrum to a specific, visually defined cell. Mapping between optical and MS-imaging data acquisition in order to assign spectra to individual cells is a technical challenge in the workflow that is critical for precise and accurate spatial imaging [72]. Two primary strategies have emerged to address this: front-end coordinate acquisition and back-end image-based registration. Several published strategies exemplifying these approaches are shown in Fig. 2, spanning post-acquisition cell segmentation (Fig. 2A) [73], fluorescence-guided acquisition (Fig. 2B) [53], and microscopy-driven region-of-interest targeting (Fig. 2C) [74].

Microscopy-based co-registration is the most common method for front-end coordinate acquisitions. This involves optical or fluorescence microscopy of targeted cells, followed by computational registration to align optical and MS coordinate systems [47, 75]. Methods such as SpaceM achieve this by acquiring high-resolution optical images of cell cultures prior to matrix deposition and MALDI analysis, then recording a post-acquisition image showing laser ablation marks [76]. The two images are aligned to extract the spectra corresponding to each segmented cell. This multimodal alignment enables single-cell metabolomic or lipidomic analysis directly from adherent cultures and has been scaled up in the “HT-SpaceM” platform [73]. Similarly, PRISM-MS introduces a “mass-guided” approach in which a low-resolution pre-scan identifies cell-containing pixels, and subsequent high-resolution scans focus on those locations, significantly increasing throughput and data quality [77]. Other methods include segmentation of multiplexed immunofluorescence images to define specific anatomical substructures based on antibody signals [74, 78]. These segmentations can then be overlaid on the MALDI IMS data, allowing for the computation of average mass spectra corresponding to each microscopically defined region [74]. These strategies are particularly effective when cells are spatially heterogeneous and cannot be physically isolated prior to analysis.

Microscopy and fluorescence-based imaging for single-cell segmentation and guidance are routinely used to define cellular morphology. Subsequently, MSI data is registered to these images for accurate cell-level interpretation, as MSI pixel data alone may not reliably define cell boundaries at small scales. In these frameworks, nuclear stains, membrane markers, and multiplexed immunofluorescence provide high-contrast morphological information for reliable segmentation. This division is particularly important for heterogeneous microenvironments where adjacent cells cannot be distinguished based on MSI signal alone. In pre-acquisition guidance workflows, fluorescence images are acquired prior to MSI and used to define regions of interest or precise cellular coordinates for targeted sampling, thereby improving acquisition efficiency. In post-acquisition co-registration, MSI datasets are computationally aligned to their fluorescence images, and ion signals are integrated to generate each single-cell profile. These approaches are often combined with multiplexed fluorescence or immunofluorescence imaging for simultaneous analysis of phenotypic markers and their untargeted molecular features such as metabolites, lipids, and glycans. Importantly, this integration addresses a main limitation of MSI at high spatial resolutions in that reducing pixel size does not inherently guarantee accurate cell assignment. Robust and accurate single-cell MSI depends on the ability to map molecular signals onto biologically defined cellular units, which can be most reliably achieved through microscopy-informed segmentation.

Image co-registration techniques primarily rely on cells adhering directly to a slide, which can limit application to weakly adherent cell types, and excludes non-adherent, suspension cell types such as circulating immune cells. Cellular therapies like CAR-T cells are increasingly being used clinically for cancer and auto-immune disease [79, 80]. Emerging evidence highlights metabolic remodeling as a primary driver of immune cell functionality as well as a predictive determinant of therapeutic success [81]. To address this gap, coordinate-based array approaches have been developed to capture and index suspension and cultured adherent cells in fixed positions [82]. These platforms represent a translational shift in the field, particularly enabling the analysis of non-adherent populations and establishing a robust foundation for pre-clinical screening assays and personalized medicine.

**Fig. 3:**
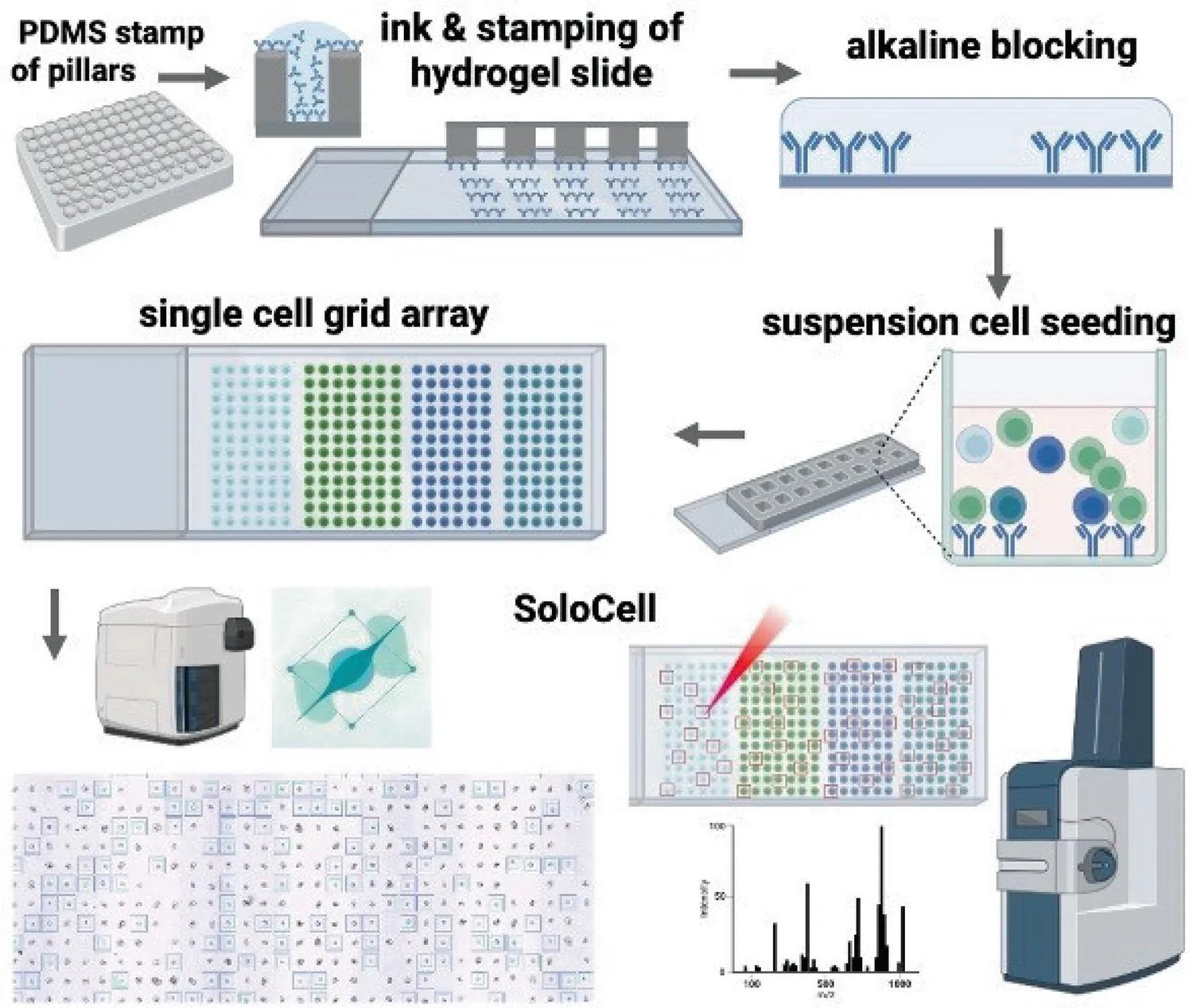
SoloCell workflow for gridded cell data acquisition. A PDMS stamp is coated with the capture antibody/lectin/bait protein and inked on the hydrogel slide. Following stamping, alkaline blocking is performed to establish the grid pattern. Cell suspensions are then incubated in chamber wells. Once the slide is washed and brightfield imaged, it is run through SoloCell for cell position identification and coordinate collection. Data is rapidly acquired at 6 cells per second allowing for thousands of cells to be captured within minutes

In coordinate-based approaches, the position of each individual cell is determined before the MSI experiment. For instance, the recent workflow reported by Dressman et al. captured individual cells in pre-defined grid locations and record their coordinates prior to MALDI imaging (Fig. 3) [83]. By knowing cell positions before data acquisition, each laser shot can be precisely targeted, avoiding mixed ablation between adjacent cells and eliminating the need for post-hoc image alignment. This front-end approach is particularly advantageous for well-spaced cells or microarrayed samples, as it simplifies downstream data analysis and allows spectra to be directly linked with cellular metadata such as phenotype or experimental condition. Furthermore, the pre-imaging allows for a multi-omic approach on these slides, enabling sequential repeat analyses to obtain a complete metabolic landscape for each identified cell [84]. Figure 3 presents an example workflow using suspension cells. Capture arrays are created using micro-contact printing with a polydimethylsiloxane (PDMS) stamp. The stamp is “inked” with an antibody solution, enabling the antibody to attach to an amine-reactive glass slide surface when the stamp is applied, mirroring the stamp configuration. Stamped slides are fitted with well chambers for the capture of multiple samples, and cell suspensions are subsequently applied and incubated. Excess cell suspension is washed away, cells are fixed to ensure adherence, and a high-resolution image of the slide is taken. The image is uploaded into a specialized software platform, such as SoloCell for this example, and selected frames are fed into a convolutional neural network trained to recognize and log the coordinates of only single cells. Once the frames are processed, the software outputs a log file containing sorted single-cell coordinates which can be uploaded into the mass spectrometer for imaging of each single cell.

**Fig. 4:**
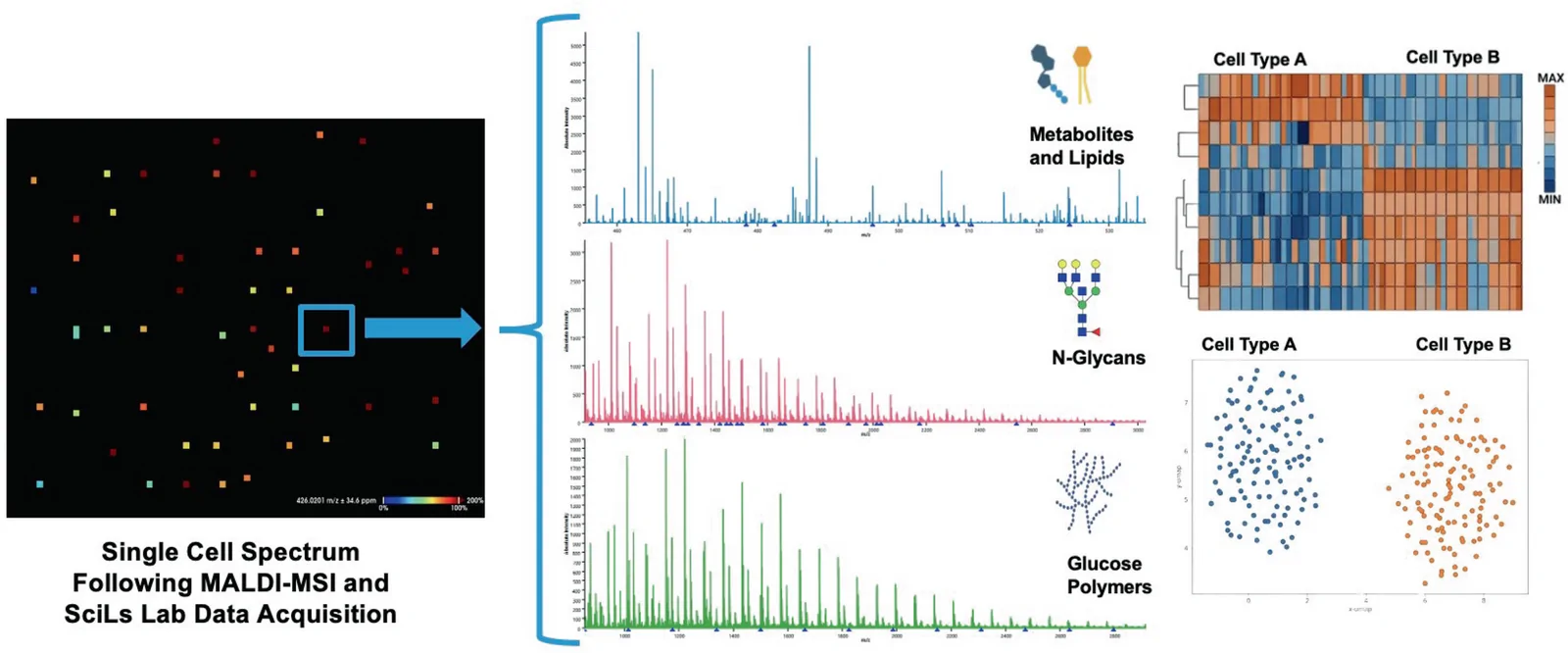
Representative image of data acquisition resulting from MALDI-MSI single-cell microarray platform. Each single cell can be visualized using SCiLS Lab software. From one single cell, one can perform sequential analysis of metabolites, lipids, N-linked glycans, and glycogen. Various software and computational analysis platforms can be used for analysis and data visualization

No matter the chosen acquisition method, single-cell MSI results in an immense data volume that quickly surpasses the capabilities of manual interpretation. As a result, dedicated software and computational analysis frameworks have become essential for data handling, normalization, and feature extraction. Platforms such as SCiLS Lab, Cardinal (R/Bioconductor), METASPACE, or Python-based pipelines have been used to perform spectral extraction, normalization, and clustering at single-cell resolution. To interpret these datasets, many groups are integrating machine learning (ML) and artificial intelligence (AI) approaches [85]. For example, the DATSIGMA framework was recently proposed as an open-source, data-driven and machine learning–based workflow specifically for processing and analyzing image-guided single-cell MS data [86]. This framework aims to enhance feature extraction and interpretability, highlighting a critical trend toward developing standardized pipelines to improve reproducibility. As illustrated in Fig. 4, the SoloCell platform enables rapid single-cell acquisition while addressing computational bottlenecks, resulting in MSI datasets for approximately 15,000 single cells to be fully acquired in ~ 40–45 min. From a single cell, application of sequential, multi-omic workflows results in a comprehensive profile of proteins, metabolites, lipids, N-linked glycans, and glycogens. Such pipelines, especially those enabling data integration on the multi-omic scale, offer a powerful approach to directly compare molecular signatures and facilitate the downstream interpretation of high-dimensional datasets.

## Conclusion and future opportunities

The significant advances in the spatial transcriptomics and multiplexed spatial proteomic fields over the past 5 years have illuminated the impact that single-cell level analysis can have for basic biology and disease processes. The molecules best suited for detection by MSI are non-templated biosynthetically and provide synergistic data when done on the same tissue/cell as the targeted transcriptomic and proteomic methods. The recent advancements in MSI instrumentation and technology have been pivotal in uncovering unique cell states and subtle molecular differences within heterogeneous cell populations. Achieving single-cell resolution with these platforms allows for physiological and pathological questions to be addressed at an unprecedented level, revealing new insights previously obscured by tissue-level averaging. MALDI-MSI offers many options for spatial single-cell profiling in terms of robustness, throughput, and sensitivity. Furthermore, using MALDI-MSI with cell capture arrays allows multi-omic profiling, enabling detection of lipids, metabolites, peptides, and glycans for thousands of individual single cells (cite Jake D paper). It should be feasible to adapt these single-cell arrays for use with SIMS and DESI-MSI applications, providing the advantages for analyte detection inherent to each ionization platform. The full potential of single-cell MSI still hinges on overcoming significant hurdles such as variability in sample preparation and data processing. These high-resolution techniques generate high-dimensional datasets, highlighting the need for specialized software and robust computational algorithms, and the field continues to move toward advancements in analytical strategies such as machine learning and sophisticated spatial statistics to unmask complex spatial and molecular patterns.

Standardization and reproducibility remain critical barriers to the clinical and translational adoption of single-cell MSI. As substantial variability persists across workflows in instrumentation, sample preparation, and data acquisition, differences in spatial resolution, ionization conditions, and analyte coverage can lead to inconsistencies in molecular identification across platforms. This variability among the single-cell MSI field highlights the need for future quantitative benchmarks and cross-study comparisons. Establishing these performance metrics and ensuring reproducibility at cellular resolution are critical directions for transitioning single-cell proof-of-concept studies to reliable, scalable, and clinically actionable workflows.

Single-cell mass spectrometry imaging represents an emerging approach for spatially resolved molecular profiling, and each method can be defined by distinct trade-offs in sensitivity, resolution, and molecular coverage. Future progress will depend on integrative, multimodal strategies that leverage complementary strengths to achieve robust and high-throughput characterization of cellular heterogeneity within complex biological systems.
